# 2180: hnRNP K overexpression drives acute myeloid leukemia emergence and progression

**DOI:** 10.1017/cts.2017.207

**Published:** 2018-05-10

**Authors:** Marisa Hornbaker, Miguel Gallardo, Xiaorui Zhang, Huaxian Ma, Peter Hu, Stephen Kornblau, Carlos Bueso-Ramos, Sean Post

## Abstract

OBJECTIVES/SPECIFIC AIMS: Acute myeloid leukemia (AML) is a devastating hematologic malignancy wherein <20% of patients will survive 5 years after diagnosis. In an effort to understand alterations that drive AML development and progression, The Cancer Genome Atlas detailed the most common recurrent mutations. One gene of interest identified here was HNRNPK, supporting our clinical observations that suggest altered expression levels of HNRNPK and its corresponding protein (hnRNP K) may impact AML. Here, we aim to elucidate the impact of hnRNP K overexpression in AML by utilizing AML cell lines and mouse models reflective of the human disease. METHODS/STUDY POPULATION: We utilized fluorescence in situ hybridization (FISH), qRT-PCR, and reverse phase protein array (RPPA) to evaluate HNRNPK copy number and expression levels in AML patient samples compared with CD34+ cells from healthy human donor bone marrow. Kaplan-Meier survival analyses were performed using clinical data from 415 AML patients at MD Anderson Cancer Center and stratified based on hnRNP K protein expression as evaluated by RPPA. To directly evaluate the impact of hnRNP K overexpression in vivo, we created 2 distinct lines of Hnrnpk transgenic mice (HnrnpkTg). Phenotypic differences in the hematologic compartments of these mice were evaluated via flow cytometry, immunohistochemistry, and transplantation assays. Molecular pathways have been evaluated in mice and cell lines using immunoblotting, qRT-PCR, and RNA-immunoprecipitation. The drug JQ1 was used in vitro with both OCI-AML3 cell lines and with primary bone marrow and splenocytes from HnrnpkTg mice. RESULTS/ANTICIPATED RESULTS: FISH analyses demonstrated that a large proportion of AML cases had amplification of HNRNPK that corresponded with upregulation of HNRNPK at the RNA and protein levels. Indeed, patients with high levels of hnRNP K had decreased overall survival compared with those expressing lower hnRNP K levels. In line with these clinical observations, we observed altered myelopoiesis in HnrnpkTg mice. These mice demonstrate increased CD11b+Gr1+ populations in the bone marrow and peripheral blood. Indeed, these mice develop myeloid leukemia, indicated by >20% of circulating white blood cells harboring markers of immature stem cells in conjunction with positive myeloperoxidase staining and blast-appearing morphology. RPPA revealed expression of c-Myc positively correlated with increased hnRNP K levels. In HnrnpkTg mice, c-Myc protein was increased, yet MYC RNA was invariably decreased compared to wildtype. To decipher a mechanism by which this may occur, we demonstrated a post-transcriptional interaction between hnRNP K and c-Myc in vivo. JQ1, a BRD4 inhibitor, that epigenetically decreases c-Myc expression showed preferential activity against myeloid cells expressing high levels of hnRNP K both in vitro and in vivo. DISCUSSION/SIGNIFICANCE OF IMPACT: These preliminary studies demonstrate that hnRNP K overexpression causes myeloid malignancies in both mouse and man. We have determined that c-Myc contributes in part to hnRNP K-mediated leukemogenesis, and that targeting c-Myc may be an effective strategy for hnRNP K-overexpressing AML. We are currently validating other potential targets for interaction with hnRNP K by performing RNA-Seq and hnRNP K immunoprecipitation followed by mass spectrometry. Fortunately, several of our putative targets are druggable—allowing for viable translational outputs to these mechanistic studies.

